# A cost-effectiveness evaluation of methods for screening for TB infection in patients with diabetes mellitus

**DOI:** 10.5588/ijtldopen.25.0311

**Published:** 2025-12-10

**Authors:** M.I. Mahmood, M. Ganason, Z.A. Aziz, S.H. Ramli, S.H.A. Halim

**Affiliations:** 1 Disease Control Division, Ministry of Health Malaysia, Putrajaya, Malaysia;; 2 National Public Health Laboratory, Ministry of Health Malaysia, Putrajaya, Malaysia.

**Keywords:** tuberculosis, Malaysia, QuantiFERON test, diabetes

## Abstract

**BACKGROUND:**

Globally, 10.6 million TB cases occurred in 2021. Malaysia reported 25,000 TB cases in 2022, and also has a high prevalence of diabetes mellitus (DM), amplifying TB risks.

**OBJECTIVE:**

Assess the cost-effectiveness of QuantiFERON (QFT-plus) versus tuberculin skin test (TST) for diagnosing TB infection in patients with DM in Malaysia.

**METHODS:**

Using an R-based model, we compared QFT-plus to TST. The analysis included individuals with HbA1C >10%. Primary outcomes encompassed costs, quality-adjusted life-years (QALYs), and averted TB cases.

**RESULTS:**

QFT-plus costs US$111.29 per person versus US$285.66 for TST. It generated 16.51 QALYs per person, compared to 16.50 QALYs for TST. Despite higher initial screening costs, its improved diagnostic accuracy enables earlier treatment, reducing active TB progression risk by 26% compared to TST. Both deterministic and probabilistic sensitivity analyses consistently favoured QFT-plus.

**CONCLUSION:**

Despite its higher initial cost, QFT-plus is both more economical and clinically superior to TST in the context of Malaysia’s high prevalence of DM. This underscores the need for its broader adoption through policy shifts to incorporate QFT-plus into national guidelines. Future research should address implementation and broader socio-economic aspects.

TB is frequently the leading infectious killer worldwide, accounting for 10.6 million global cases and resulting in 1.13 million deaths in 2022.^1^ Beyond active TB cases, TB infection (TBI), formerly known as ‘latent TB’, is estimated to be present in 1.7 billion people.^2^ TBI refers to a state where the *Mycobacterium tuberculosis* bacteria are present in the body without causing symptoms or being contagious. In contrast, active TB occurs when the infection progresses, leading to clinical symptoms and the potential for transmission.^3^ Malaysia, a medium-TB-burden country, has an estimated incidence of 77.8 cases per 100,000 population. In 2022, 25,391 cases were reported, 17% rise compared to 2021. Another leading killer, diabetes mellitus (DM), triples the risk of active TB, making it challenging to control and reduce TB disease and infection without simultaneously addressing these overlapping epidemics.^4^ In 2021, 537 million adults were living with DM worldwide, with over three out of four residing in low- and middle-income countries.^5^ This number is expected to rise to 643 million by 2030 and 783 million by 2045. In 2021, DM accounted for 6.7 million deaths.^5^ The prevalence of DM in Malaysia increased from 11.2% in 2011 to 18.3% in 2019, affecting about 3.9 million Malaysian adults.^6^ This dual epidemic requires a comprehensive, strategic approach for screening individuals and communities for TBI, before the development and transmission of active TB disease. The interrelationship between DM and TB is increasingly evident and concerning. DM modulates the immune system such that it amplifies susceptibility to infections. Notably, patients with diabetes exhibit a heightened probability of transitioning from TBI to an active TB state.^4^ Recent studies suggest not only overlap of these diseases in certain populations but also some underlying mechanistic relationship between DM and TBI. A systematic review showed that people with DM were 1.18 times more likely to acquire TBI.^7^ A study in the United States (U.S.) that screened refugees for DM found that people with pre-DM and DM had a higher chance of having TBI.^8^ Similarly, a study from Taiwan found that people with DM showed a higher risk of TBI.^9^ In another U.S. study, people with TBI were more often found to have DM compared to those without TBI.^10^ The dual epidemics of TB and DM in Malaysia are expected to cause a surge in TB cases; thus, implementing a cost-effective and robust TBI screening programme is critical. Given the difficulty in controlling diabetes in individual patients in many settings, a robust TBI screening approach is potentially transformative for Malaysia to reduce mortality among the group suffering from this dual epidemic.

The tuberculin skin test (TST) is commonly used to diagnose TBI, but its accuracy can be affected by cross-reactivity with the Bacille Calmette-Guérin (BCG) vaccine and certain non-TB mycobacteria.^11^ TST involves injecting a small amount of purified protein derivative tuberculin into the skin and measuring the size of the resulting induration after 48–72 h, which indicates an immune response. For TST, sensitivity is estimated at 77% and specificity varies: 59% in BCG-vaccinated individuals and 97% in non-BCG-vaccinated individuals.^12^ Interferon-gamma release assays (IGRAs), like QuantiFERON-TB Gold Plus (QFT-plus), T-SPOT, and WANTAI TB-IGRA, are suitable alternatives, offering high sensitivity and specificity in detecting TBI. QFT-plus is a blood-based assay that measures interferon-gamma concentration using an enzyme-linked immunosorbent assay (ELISA) after stimulating T-cells with specific *M. tuberculosis* antigens. It provides objective results and is unaffected by BCG vaccination or exposure to most non-TB mycobacteria.^12–14^ QFT-plus demonstrates higher sensitivity (91%) and consistent specificity (97%), irrespective of BCG vaccination status.^15^

There is limited consensus on screening strategy for TBI in people with DM, both globally and in Malaysia. It remains unclear which TBI screening test provides the most cost-effective approach for screening and treating individuals with diabetes in Malaysia. Recent studies suggest that the comparatively low cost of utilising TST (due to its not requiring a laboratory setup) is outweighed by its limitations, compared to IGRAs, at a relatively low population threshold in middle-income settings, even accounting for the cost of the laboratory.^16^ Thus, this study estimated the cost-effectiveness of QFT-plus compared to TST for the population with diabetes in Malaysia.

## METHODS

The economic model, integrating a decision tree and a Markov model, evaluated the effectiveness of QFT-plus with TST from Malaysia’s health care perspective.^17^ The decision tree captured immediate outcomes of TBI screening, such as test results and treatment initiation, while the Markov model projected long-term health and economic outcomes. The Markov model included key health states: ‘no active TB’, ‘active TB’, and ‘post-TB treatment’, with transitions reflecting the natural progression of TBI and the effects of interventions. The model simulates transitions between different TB-related health states based on treatment effectiveness, adverse events, and reactivation rates. Healthy individuals remain in the healthy state without transitions. Untreated, treatment-naïve TBI may progress to active TB based on reactivation rates (estimated at 0.1% annually) but cannot directly transition to treatment states. This annual probability of progression reflects a lifetime average reactivation risk, rather than a time-varying hazard function. While TB progression risk is known to be highest in the first 2 years after infection and then decline, modelling this dynamic directly would require a more complex structure. Instead, our approach assumes a constant risk, which is computationally feasible and suitable for a population in which the timing of infection varies. The model applies a 3% annual discount rate, which naturally gives more weight to earlier TB cases. Furthermore, both deterministic sensitivity analysis (DSA) and probabilistic sensitivity analysis (PSA) were conducted to test the robustness of the model across a wide range of progression rates. This ensures that the model’s conclusions are not sensitive to the assumption of a constant reactivation rate. Individuals receiving TBI treatment (6-month daily isoniazid), with or without adverse events, may return to health or become previously treated TBI, depending on treatment success. Previously treated TBI remains stable unless reactivation occurs. Active drug-sensitive or multidrug-resistant TB resolves with treatment, returning individuals to the healthy state with 100% probability, reflecting successful therapy outcomes. Transition probabilities were derived from national datasets, published studies, and expert input, incorporating factors such as TBI reactivation, treatment success, and mortality (e.g., a 5% case fatality rate for active TB). Quality-adjusted life-years (QALYs) were calculated by applying utility weights to each health state, adjusted for TB-related morbidity and mortality, and aggregated over the model’s time horizon. Costs and outcomes, encompassing both direct and indirect health care costs, were discounted at an annual rate of 3%. This analysis focused on individuals with HbA1c >10%. This threshold was selected to model a feasible and realistic implementation scenario, given the operational and resource constraints faced by Malaysia’s health care system. While HbA1c >6.5% is used for diabetes diagnosis, targeting individuals with HbA1c >10% identifies a subgroup with poorer glycaemic control and higher TB progression risk, allowing for a more manageable, high-risk group for initial screening scale-up. A detailed summary of parameters and data sources is provided in Tables 1 and 2. Although the model incorporated TB’s mortality risk, adverse event mortality was set at 0% due to limited evidence.

**Table 1.** Model inputs.

Parameter	Value	Standard error	PSA distribution	Reference
Population size	141,745	N/A	N/A	18
Average age	53 years	N/A	N/A	18
Proportion, male	0.429	N/A	N/A	18
TBI prevalence	0.114	0.020	Beta	19
All-cause mortality	Age-dependant	N/A	N/A	1
DS-TB mortality risk	0.125	0.012	Beta	20
MDR-TB mortality risk	0.375	0.037	Beta	20
Treatment efficacy	0.69	0.069	Beta	15
Adherence rate	0.565	0.056	Beta	21
Probability of AE (drug-related hepatotoxicity)	0.452	0.045	Beta	22
Initial reactivation rate (treatment naive)	0.0079	0.007	Beta	23
Initial reactivation rate (previously treated)	0.0017	0.17	Beta	23
Proportion of MDR-TB cases (treatment naive)	0.012	0.001	Beta	1
Proportion of MDR-TB cases (previously treated)	0.031	0.003	Beta	
Proportion BCG-vaccinated	0.99	N/A	N/A	24
Sensitivity of TST	0.77	0.03	Beta	12
Specificity of TST (BCG-vaccinated)	0.59	0.07	Beta	
Specificity of TST (non-BCG-vaccinated)	0.97	0.03	Beta	
Sensitivity of QuantiFERON	0.91	0.03	Beta	15
Specificity of QuantiFERON	0.97	0.01	Beta	
Healthy	1	N/A	N/A	25,26
Untreated TBI	1	N/A	N/A	
TBI, taking treatment without AE	0.99	0.099	Beta	
TBI, taking treatment with AE	0.85	0.085	Beta	
DS-TB	0.8	0.08	Beta	
MDR-TB	0.58	0.058	Beta	

TBI = TB infection; AE = adverse events; DS-TB = drug-susceptible TB; MDR-TB = multidrug-resistant TB; TST = tuberculin skin test; BCG = Bacillus Calmette-Guérin.

**Table 2.** Unit costs.

Parameter	Description	Cost (USD)	Source
TBI treatment	The average cost of TBI treatment per patient.	49.5 per patient	GDF
DS-TB treatment	The average cost of treating drug-sensitive TB per patient.	68.90 per patient	GDF
MDR-TB treatment	The average cost of treating MDR-TB per patient.	927.38 per patient	GDF
QuantiFERON	The cost of a QuantiFERON test for one patient.	20.39 per test	GDF
TST	The cost of a TST for one patient.	2.40 per test	Country specific
Health care worker contact time			
Nurse time	The cost of a nurse per hour.	3.6 per hour	Country specific
Hospital physician time	The cost of a hospital physician per hour.	47.97 per hour	Country specific
General practitioner	The cost of a general practitioner per hour.	19.19 per hour	Country specific
TB specialist time	The cost of a TB specialist per hour.	47.97 per hour	Country specific
DOT sessions	The cost of a DOT session.	3.6 per session	Country specific
Environmental officer	The cost of an environmental health officer per hour		Country specific
Diagnostic tests			
Smear test of sputum	The cost of smear tests per patient.	0.32 per test	GDF
Culture of sputum examination	The cost of culture tests per patient.	6.70 per test	GDF
Chest X-ray	The cost of chest X-rays per patient.	14.39 per test	Country specific
ESR	The cost of ESRs per patient.	14.39 per test	Country specific
GeneXpert (PCR test)	The cost of GeneXpert tests to check for drug sensitivity per patient.	9.98 per test	GDF
Laboratory technician time	The cost of a laboratory technician per hour.	6 per hour	Country specific
Adverse events			
Liver function test	The number of liver function tests per patient.	14.39 per test	Country specific
Laboratory technician time	The cost of a laboratory technician per hour.	6 per hour	Country specific
Treatment of drug-induced hepatotoxicity	The average cost of treatments per patient.	836.6 per test	Country specific
Active TB case contacts			
Contacts per active case	The average cost associated with contacts per active case of TB.	9.59 per case	Country specific
Other			
Hospitalisation	The average cost of hospitalisation per day.	47.97 per day	Country specific

TBI = TB infection; AE = adverse events; GDF = Global Drug Facility; DS-TB = drug-susceptible TB; MDR-TB = multidrug-resistant TB; TST = tuberculin skin test; ESR = erythrocyte sedimentation rate; DOT = directly observed therapy; PCR = polymerase chain reaction.

Both DSA and PSA were conducted to test the robustness of model conclusions. DSA varied key parameters one at a time (e.g., test sensitivity, specificity, and costs) across plausible ranges to assess their impact on results. PSA used 5,000 Monte Carlo simulations, sampling from probability distributions (beta for sensitivity/specificity, gamma for costs) to capture parameter uncertainty in a probabilistic framework. These methods help ensure the conclusions are robust across varying assumptions.

### Ethical statement

No ethical approval was required, as no human datasets were generated or analysed.

## RESULTS

The main results are summarised in Tables 1, 3, and 4. The overall costs for each individual were US$285.66 for TST and US$111.29 for QFT-plus. QALY outcomes were also favourable for QFT-plus, with an average of 16.51 QALYs per person compared to 16.50 QALYs for TST. This incremental gain, though modest, contributes to the favourable cost-effectiveness profile of QFT-plus when combined with cost savings and reduced disease burden. Even though QFT-plus screening is more expensive – at US$25.19 compared to US$4.20 for TST – QFT-plus saves money in other areas (Table 3). The lifetime cost-effectiveness analysis demonstrates that, despite higher initial screening costs, QFT-plus results in overall lower costs per patient compared to TST (US$111.29 vs. US$285.66).

**Table 3.** Discounted costs breakdown (per patient) for QuantiFERON versus tuberculin skin test.

Item	QuantiFERON cost (USD)	Tuberculin skin test cost (USD)
Screening	25.19	4.20
Treatment	4.04	13.07
Health care worker contact time	40.45	132.07
TB infection adverse events	39.68	133.71
Diagnosis tests	1.05	1.42
Active TB case contacts	0.48	0.64
Other	0.41	0.55
Total	111.29	285.66

**Table 4.** Active TB cases avoided through use of QuantiFERON versus tuberculin skin test.

Item	QuantiFERON	Tuberculin skin test
Lifetime active TB rate per patient	0.0069	0.0093
Cost per avoided active TB case (USD)		−71,545
Number needed to treat		410

This cost difference arises due to QFT-plus’s superior sensitivity (91% vs. 77% for TST) and specificity (97% vs. 59% in BCG-vaccinated individuals), which lead to fewer missed cases of TBI and fewer false positives. These advantages translate into reduced progression to active TB (0.0069 per patient for QFT-plus vs. 0.0093 for TST) (Table 3), facilitating earlier and more effective preventive treatment. Additionally, fewer unnecessary treatments reduce health care worker contact time and adverse event costs, as detailed in Table 1. Furthermore, reduced health care worker contact time (US$40.45 vs. US$132.07 for TST) and lower adverse event costs (US$39.68 vs. US$133.71 for TST) significantly contribute to the overall savings. By preventing more cases of active TB while lowering associated costs, QFT-plus is a more cost-effective choice over a patient’s lifetime to manage Malaysia’s dual TB-diabetes epidemic. This model suggests that using QFT-plus could lower the chances of someone with TBI developing active TB (Table 4). With TST, the risk of getting active TB increases by about 26% compared to using QFT-plus. To prevent one additional case of active TB compared to TST, a total of 410 patients need to be screened with QFT, resulting in a cost saving of US$71,545 per active TB case for QFT versus TST.

Both DSA and PSA were performed. The DSA showed that QFT-plus remained cost-effective across a wide range of input parameters, including reactivation rates, test costs, and test accuracy assumptions. The PSA, conducted with 5,000 Monte Carlo simulations using beta and gamma distributions for key variables, confirmed that QFT-plus was cost-effective in nearly all iterations.

## DISCUSSION

The aim of the study was to estimate the cost-effectiveness of QFT-plus compared to TST for the population with diabetes in Malaysia. The results consistently showed that QFT-plus was cost-effective compared to TST across a wide range of willingness-to-pay thresholds. This indicates that the conclusion of QFT-plus being the more cost-effective option remains stable even when accounting for uncertainties in model inputs.

The cost-effectiveness analysis is crucial for promoting the rapid adoption of TBI screening in specific at-risk populations, especially in countries with a medium TB burden, such as Malaysia, with the resources and laboratory capacity to utilise QFT-plus reliably. Although QFT-plus has higher initial setup and screening costs compared to TST, it offers substantial overall cost savings in patient management. Notably, despite increased upfront screening expenses, the cumulative expenditure for QFT-plus remains substantially lower than TST at any reasonable expected levels of utilisation. This underscores the significant indirect (hidden) costs associated with sub-optimal screening methods and the need for comprehensive cost-effectiveness assessment when evaluating interventions. The lesser sensitivity and specificity of TST may lead to more false-negative and false-positive results, with potential consequences of undiagnosed TB cases, over-treatment, and associated wasted resources and effort. Moreover, given the significant prevalence of DM in Malaysia and its association with increased risk of TBI, efficient screening and management strategies are urgently needed. By targeting populations with HbA1C >10%, this study sheds light on a particularly at-risk subset of patients with diabetes; there could be broader ramifications for other diabetes cohorts.

The distinct variations observed in the probabilities of active TB manifestation between the TST and QFT-plus methods within this model underscore the imperative to rigorously evaluate and adopt the most efficient screening methodology in this group. Malaysia’s escalating burden of DM and TB, and the known immunosuppressive effects of DM, makes this differential not merely a financial consideration but an urgent clinical and ethical one. The severe consequences of active TB – especially among those with compromised immune functionality (e.g., diabetes) – strengthen the impetus to minimise this risk.

Fortunately, the global TB screening paradigm is shifting. Because TB has long been a disease of poverty worldwide, screening approaches have been comparatively poorly funded relative to its true impact on morbidity and mortality. These antiquated approaches largely revolved – and continue to revolve in many countries – around passive case finding and limited TBI screening. After the 2018 UN High-Level Meeting on TB, the approach to screening for TBI and active TB has been updated to better align with current understanding of how the global ‘reservoir’ of TBI progresses to active disease.^27^ Signatory member states acknowledged people living with diabetes as a vulnerable group at increased risk of TB. The Political Declaration commits to systematic screening of relevant risk groups, including people with diabetes, for both active and latent TB. While not listed among the primary targets for preventive treatment, diabetes is clearly recognised as a major co-morbidity in the TB response, underscoring the need for targeted interventions in this group. Malaysia is committed politically to this effort and will work to reach its share of the agreed-upon TBI and TB disease screening targets, while also considering this critical challenge of DM in its hardest-hit communities.

While QFT testing is already in use in Malaysia for selected groups such as health care workers and migrant populations, expanding its use to people with diabetes introduces new operational challenges. These include adapting sample collection and transport workflows to non-communicable disease (NCD) clinics, training providers unfamiliar with TB diagnostics, and ensuring follow-up of QFT-positive patients. The challenge is not the novelty of QFT, but the need for care integration, especially where diabetes care is managed outside traditional TB settings. Without formal guidance or reimbursement pathways for TBI screening in diabetes, the use of QFT in this population remains limited despite existing infrastructure. Table 5 summarises these context-specific barriers.

**Table 5.** Implementation challenges for QuantiFERON (QFT)-based TBI screening in people with diabetes mellitus (DM).

Domain	Challenge	Relevance to QFT implementation	Contextual notes for Malaysia
Service integration	Fragmentation between TB and DM/NCD clinics	QFT is mostly used in TB or occupational health settings; not routinely integrated with diabetes care	QFT infrastructure exists, but expansion into non-communicable disease (NCD) clinics is not yet standardised
Diagnostic logistics	Expanding QFT logistics to new care settings (e.g., DM clinics)	Requires extending sample collection and transport workflows to additional patient groups	Sample transport and test coordination outside TB clinics remain a practical barrier
Lab infrastructure	Uneven QFT access at lower-tier or rural facilities	Existing QFT labs serve urban areas and specific populations (e.g., health care workers and migrants)	Further rollout needed to reach general diabetes patients, especially outside TB-designated facilities
Policy framework	Lack of explicit guidance on TBI testing for people with DM	QFT availability does not guarantee use unless aligned with clinical or national screening policies	DM is not currently prioritised for routine TBI testing in national TB guidelines

In the context of TB screening for patients with diabetes in Malaysia, our findings underscore clear economic advantages of the QFT-plus methodology over TST and the well-known operational and performance benefits of IGRA methods in general. It is imperative, especially for low-/middle-income countries’ health systems, to adapt to this emerging evidence and move towards more cost-effective strategies for addressing such intertwined epidemics. By making this shift, we can expect not just increased efficiency of health care spending but enhanced health outcomes for the population at large. This study’s findings can serve as a catalyst for decision-makers to accelerate the integration of TBI screening using IGRAs into their national guidelines. Such integration is essential, considering Malaysia’s distinct epidemiological challenges with both DM and TB. Future research should delve into the practicalities of implementation and the broader socio-economic aspects of transitioning to more active and effective TBI screening methods.
